# Lymphocytes: Versatile Participants in Acute Kidney Injury and Progression to Chronic Kidney Disease

**DOI:** 10.3389/fphys.2021.729084

**Published:** 2021-09-20

**Authors:** Chujin Cao, Ying Yao, Rui Zeng

**Affiliations:** Division of Nephrology, Tongji Hospital, Tongji Medical College, Huazhong University of Science and Technology, Wuhan, China

**Keywords:** lymphocytes, acute kidney injury, tubular cell damage, renal fibrosis, chronic kidney disease, gut microbiota

## Abstract

**Background:** Acute kidney injury (AKI) remains a major global public health concern due to its high morbidity and mortality. The progression from AKI to chronic kidney disease (CKD) makes it a scientific problem to be solved. However, it is with lack of effective treatments.

**Summary:** Both innate and adaptive immune systems participate in the inflammatory process during AKI, and excessive or dysregulated immune responses play a pathogenic role in renal fibrosis, which is an important hallmark of CKD. Studies on the pathogenesis of AKI and CKD have clarified that renal injury induces the production of various chemokines by renal parenchyma cells or resident immune cells, which recruits multiple-subtype lymphocytes in circulation. Some infiltrated lymphocytes exacerbate injury by proinflammatory cytokine production, cytotoxicity, and interaction with renal resident cells, which constructs the inflammatory environment and induces further injury, even death of renal parenchyma cells. Others promote tissue repair by producing protective cytokines. In this review, we outline the diversity of these lymphocytes and their mechanisms to regulate the whole pathogenic stages of AKI and CKD; discuss the chronological responses and the plasticity of lymphocytes related to AKI and CKD progression; and introduce the potential therapies targeting lymphocytes of AKI and CKD, including the interventions of chemokines, cytokines, and lymphocyte frequency regulation *in vivo*, adaptive transfer of ex-expanded lymphocytes, and the treatments of gut microbiota or metabolite regulations based on gut-kidney axis.

**Key Message:** In the process of AKI and CKD, T helper (Th) cells, innate, and innate-like lymphocytes exert mainly pathogenic roles, while double-negative T (DNT) cells and regulatory T cells (Tregs) are confirmed to be protective. Understanding the mechanisms by which lymphocytes mediate renal injury and renal fibrosis is necessary to promote the development of specific therapeutic strategies to protect from AKI and prevent the progression of CKD.

## Introduction

Despite the identification of clinical diagnosis and application of dialysis, acute kidney injury (AKI) remains a major global public health concern due to high morbidity and mortality with few systematic efforts to manage it including prevention, diagnosis, and treatments (Mehta et al., 2015). Rodent models of AKI have provided novel insights into the potential pathophysiologic mechanisms, which include hypoxia, oxidative stress, endoplasmic reticulum stress, mitochondrial dysfunction, and inflammation. Hypoxia and oxidative stress induce microvascular endothelium injury and endothelial cell activation with expression change of new markers, which promote the recruitment of inflammatory cells. Resident dendritic cells and macrophages initiate inflammation in response to renal injury. Subsequently, neutrophils and monocytes, which are recruited by chemotactic signals, amplify the inflammation after acute injury. Whereas, lymphocytes, especially T cells, are involved in the whole evolution of injury (Zuk and Bonventre, 2016).

With acute injury, adaptive responses restore cell, and renal tissue homeostasis. However, dysregulated or insufficient repairs impair the regeneration and contribute to chronic kidney disease (CKD) (Zuk and Bonventre, 2016; D'Alessio et al., 2019). Immune cells with high plasticity and diversity participate in almost all the events involved from renal injury to repair and the subsequent fibrosis. For example, the phenotypes of macrophages exert distinct functions in different phases of injury, including M1 proinflammatory cells in the phase of injury and M2 anti-inflammatory cells in the phase of recovery (Huen and Cantley, 2017; Tang et al., 2019). Similarly, lymphocytes possess polytropic subtypes ensure to provide precise and comprehensive regulation of immune response maintenance in injured kidney.

Generally, lymphocytes contain two major categories that are T cells and B cells. T cells, which originate from bone marrow (BM) progenitors, migrate to the thymus for maturation and subsequently export into the periphery, are divided into alpha beta T (αβT) cells and gamma delta T (γδT) cells according to respective T-cell receptors (TCRs) on their surfaces. αβT cells are further classified into cluster of differentiation (CD)4^+^T cells, CD8^+^T cells, and double-negative T (DNT) cells, of which naïve CD4^+^ T cells differentiate into various helper subsets in immune responses such as T helper (Th)1, Th2, and Th17. In addition, one unique type of T cells, called regulatory T cells (Tregs), plays an important role in immune tolerance and homeostasis (Kumar et al., 2018; Zhu, 2018).

Another type of lymphocytes, named innate lymphoid cells (ILCs), have recently seen a great upsurge in studies related to kidney diseases. Unlike T cells and B cells, ILCs lack diversified and adaptive antigen receptors, which determine their innate-immune properties. ILC1, ILC2, and ILC3 are the innate counterparts of Th1, Th2, and Th17, respectively (Vivier et al., 2018). Besides ILCs, several types of T cells function like innate cells and exist extensively in normal kidneys with tissue-resident characteristics, including invariant natural killer T (iNKT) cells, mucosa-associated invariant T (MAIT) cells, and γδT cells. These innate or innate-like lymphoid cells respond earlier to renal damage than adaptive lymphocytes (Turner et al., 2018).

Recent studies have challenged the view that CD4^+^ T helper cell subsets are a cluster of terminally differentiated homogeneous cells, demonstrating that T cells have more powerful plasticity than previously thought. Ulf Panzer et al. summarized the current perceptions of Th17 cell plasticity and heterogeneity in autoimmune kidney diseases and debated the single-side and harmful effect of Th17 cells on renal inflammation (Krebs and Panzer, 2018). In addition, the stability and anti-inflammatory effect of Tregs are also in doubt. Researchers found that with the acquisition of hybrid fates, Tregs became unstable under certain inflammatory conditions and exerted promotion rather than suppression of inflammation (Sakaguchi et al., 2013). Furthermore, ILCs and γδT cells are also flexible in the inflammatory milieu with diverse activating signals (Corpuz et al., 2017; Colonna, 2018). Understanding the molecular basis of lymphocyte heterogeneity and plasticity in AKI and CKD may allow the development of therapies to target lymphocytes in a specific manner.

In this review, we summarized the mechanisms by which various types of lymphocytes participate in AKI, subsequent repair and progression to CKD with a focus on T cells and ILCs. Then, we illuminated the diversity and plasticity of these cells with time-course to progression and inflammatory status changes. Finally, we discussed the potential effective therapeutic interventions associated with lymphocytes for AKI and CKD under current studies.

## Lymphocytes Mediate AKI and CKD: Diversity and Mechanisms

**Figure 1:**
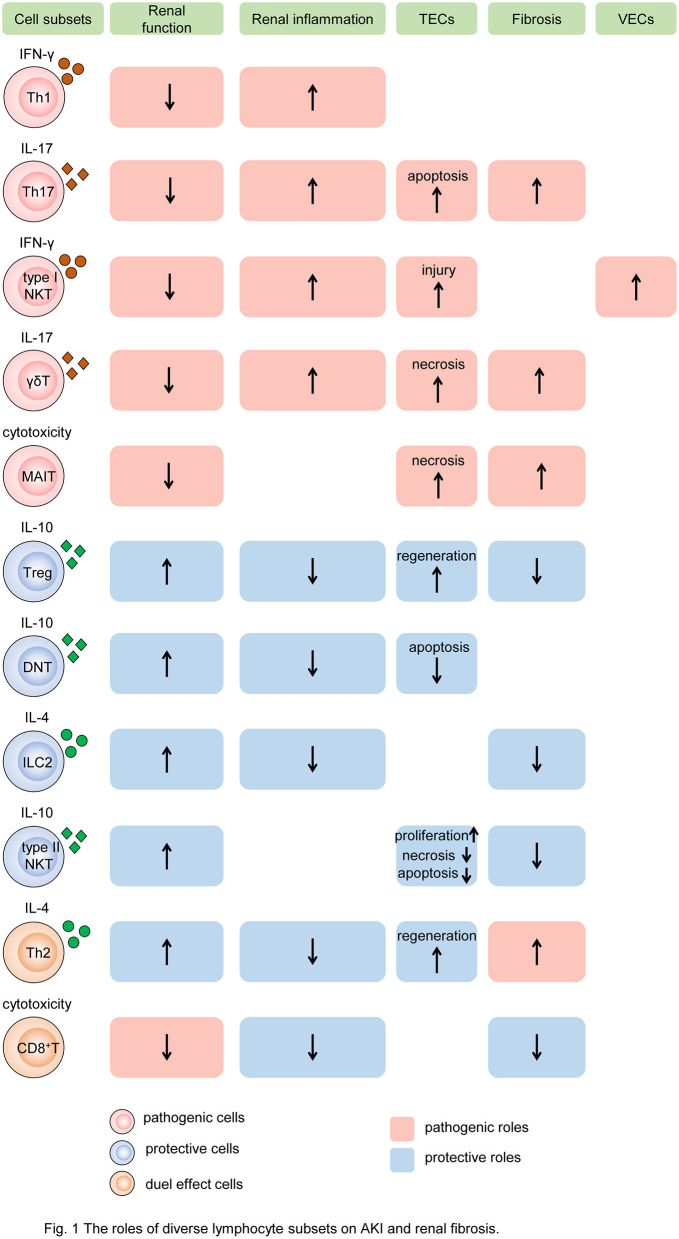
The roles of diverse lymphocyte subsets on AKI and renal fibrosis. Different subsets of T cells, innate-like lymphocytes, and innate lymphoid cells mediate renal injury and fibrosis by regulating intrinsic cell death, proliferation, and fibroblast formation. In the process, cytokine secretion and cytotoxicity are two major mechanisms. Some subsets of lymphocytes, such as Th1, Th17, type I NKT cells, and γδT cells, produce pathogenic cytokines like IFN-γ, induce renal inflammatory, and impair TECs and VECs, resulting in the decline of renal function and exacerbation of renal fibrosis. While MAIT cells play the pathogenic role by cytotoxicity. Other subtypes, such as Tregs, DNT cells, ILC2, and type II NKT cells, produce protective cytokines like IL-10, reduce renal inflammation, promote TEC repair, leading to preserve renal function and alleviate renal fibrosis. In addition, Th2 and CD8^+^ T cells have dual roles in AKI and CKD. Cells colored red represent that they are pathogenic. Cells colored blue represent that they are protective. Cells colored orange represent that they have dual roles. The rectangle with red background represents the pathogenic role and that colored blue means the protective role. The up arrows mean increased roles, and the down arrows mean decreased roles. Th, T helper cell; NKT, natural killer cell; γδT, γδ^+^T cell; MAIT, mucosa-associated invariant T cell; Treg, regulatory T cell; DNT, double-negative T cell; ILC2, type 2 innate lymphoid cell; MAIT, mucosa-associated invariant T cell; IFN-γ, interferon γ; IL, interleukin; TEC, tubular epithelial cell; VEC, vascular endothelial cell.

During the past decades, many studies have uncovered that lymphocytes, particularly diverse T cells and ILCs, play a crucial role in postischemic, nephrotoxic, septic, and postrenal AKI as well as subsequent repair and CKD, which are nonautoimmune diseases (shown in Figure 1). Ischemia and reperfusion induce sterile inflammation, in which hypoxia-induced sterile cell death or injury causes the release of some ligands, leading to immune responses. Such ligands, called damage-associated molecular patterns (DAMPs), are normally detained intracellularly. However, upon tissue damage, they are released into the extracellular environment where immune responses are activated (Eltzschig and Eckle, 2011). The mechanisms of nephrotoxic AKI have a difference. Due to high blood flow and local metabolism of drugs, the kidneys are extremely sensitive to drug hypersensitivity. Drugs acting as prohaptens or haptens turn native renal proteins into neo-antigens, activating innate immune responses. And in the effector phase, nephrotoxic AKI is characterized by the infiltration of lymphocytes in the kidney (Perazella, 2019). In addition, sepsis triggers a systemic and activated immune response followed by immune suppression that may make septic AKI more severe than non-septic AKI (Alobaidi et al., 2015). Due to the multiple characteristics and functions of lymphocytes, mechanisms are intricate, by which these cells give full play to their own expertise and interact with infiltrated or intrinsic cells in kidneys. The systematical findings in animal experiments and human studies of AKI are shown in Tables 1, 2, respectively.

**Table 1:** The systematically findings of lymphocytes in animal experiments of AKI.

**Year**	**Models**	**Findings**	**References**
2001	IR	1. Mice with deficiency in CD4+ T cells, rather than those with deficiency in CD8+ T cells, were remarkedly protected from AKI that was called acute kidney failure (ARF). 2. Adaptive transfer of wild-type CD4+ T cells for the reconstitution of CD4-deficient mice was found to restore post-ischemic kidney injury.	Burne et al., 2001
2000	Cisplatin-induced AKI	Harmful role of CD4+ T cells was confirmed in murine acute cisplatin nephrotoxicity by adaptive transfer experiments.	Rabb et al., 2000
2003	IR	STAT4 deficient in mice mildly improved renal function, whereas STAT6 deficient markedly aggravated function and tubular injury.	Yokota et al., 2003
2015	IR	Activated T cells, mostly positive for IL-17, were increased in the kidney after AKI and elevated salt dietary intervention.	Mehrotra et al., 2015
2006	IR	Isolation and transfer of T lymphocytes infiltrated in kidney into T cell-deficient mice with renal IRI, reduced the functional and histological injury, thus, suggesting the possible existence of reno-protective T cell populations, Tregs.	Ascon et al., 2006
2009	IR	1. There was a significant recruitment of Tregs into kidneys 3- and 10-days post ischemia. 2. These infiltrated Tregs promoted tubular proliferation and reduced pro-inflammatory cytokine generation. 3. Depletion of Tregs worsen renal function and mortality.	Gandolfo et al., 2009
2018	FA-induced AKI	The strong upregulation of CCL20 was confirmed at day 2 of renal injury and persisted for 7 days.	Gonzalez-Guerrero et al., 2018
2009	IR	Transfer of wild-type Tregs into immunodeficient mice prevented renal IRI, but transfer of IL-10-deficient Tregs did not.	Kinsey et al., 2009
2016	IR	DNT cells expand significantly and become the dominant subsets of the early responders.	Martina et al., 2016
2020	Cisplatin-induced AKI	DNT cells alleviate cisplatin-induced dysfunction and structure damage from AKI by reducing apoptosis in kidney proximal tubular epithelial cells (PTECs).	Gong et al., 2020
2015	IR	Given the protective role of Th2 and type 2 immunity on AKI, researchers intent to whether ILC2s are also beneficial to kidney injury.	Huang et al., 2015
2019	IR	A loss of ILC2s does not alter the severity of IR-induced renal injury suggesting the redundancy of ILC2s for IRI protection.	Cameron et al., 2019b
2018	α-GalCer-induced AKI	NKT cells injured kidney vascular endothelial cells by perforin-mediated pathway and tubular epithelial cells by TNF-α/FasL pathway, leading to AKI with hematuria in mice.	Uchida et al., 2018
2014	Cisplatin-induced AKI	Depletion of γδT cells did not ameliorate cisplatin-induced renal injury indicating γδT cells were unnecessary to injury.	Chan et al., 2014

**Table 2:** The systematically findings of lymphocytes in human studies of AKI.

**Year**	**Patients**	**Findings**	**References**
2014	23 patients with sepsis	CD4+ lymphocyte adenosine triphosphate (ATP) might be a new marker in sepsis-associated AKI.	Patschan et al., 2014
2019	AKI after cardiac surgery	Th1-induced IFN-γ, Th2-induced IL-4, and IL-13 increased after surgery associated with postoperative AKI.	Moledina et al., 2019
2018	AKI secondary to glomerular injury (IgA nephropathy) diagnosed based on renal biopsy	CCL20 was increased in human kidneys and urine with AKI and urinary CCL20 was associated with severity.	Gonzalez-Guerrero et al., 2018
2015	Sepsis-associated AKI	The ratios of Tregs in peripheral blood might provide a potential biomarker to accurately evaluate prognosis of sepsis.	Chen et al., 2015
2018	20 consecutive patients undergoing multibranched endovascular thoracoabdominal aortic repair	The changes of infiltrated γδT cells were correlated with the elevated biomarkers of tubular stress or injury.	Gocze et al., 2018

### T Helper Cells

An article published in 2001 provided direct evidence of the pathogenic role of CD4^+^ T cells in AKI induced by ischemia reperfusion (IR). Researchers found that mice with deficiency in CD4^+^ T cells, rather than those with deficiency in CD8^+^ T cells, were remarkedly protected from AKI that was called acute kidney failure (ARF) at that time. The following adaptive transfer of wild-type CD4^+^ T cells for the reconstitution of CD4-deficient mice was found to restore postischemic kidney injury. In addition, the reconstitutions with CD4^+^ T cells lacking the ability of interferon gamma (IFN-γ) production were insufficient to restore kidney injury, which implied IFN-γ-producing CD4^+^ T cell might be a pathogenic factor in AKI (Burne et al., 2001). A similar harmful role of CD4^+^ T cells was confirmed in murine acute cisplatin nephrotoxicity by adaptive transfer experiments (Rabb et al., 2000). In addition, a clinical research showed that CD4^+^ lymphocyte ATP might be a new marker in sepsis-associated AKI because of its correlation with survival in sepsis (Patschan et al., 2014). In summary, CD4^+^ T cells are generally pathogenic agents in AKI induced by IR, nephrotoxic drugs, and sepsis.

Furthermore, depletion of CD4^+^ T cells was proved to retard UUO-induced renal fibrosis (Liu et al., 2012), and reconstitution of lymphopenic recombination activating gene (RAG)^−/−^ mice with CD4^+^ T cells but not CD8^+^ T cells prior to unilateral ureteric obstruction (UUO) led to more severe renal fibrosis manifesting a significant increase in interstitial expansion and collagen deposition, which revealed the pivotal role of CD4^+^ T cells in renal fibrosis (Tapmeier et al., 2010). Naïve CD4^+^ T cells activated by renal damage signals differentiate into distinct Th cells producing lineage-specific cytokines. How various subtypes of Th cells regulate the process of AKI and renal fibrosis is noteworthy.

### T Helper 1 and T Helper 2

T helper (Th) 1 and T helper 2 were initially Th cell subsets reported to preferentially produce IFN-γ and IL-4, respectively. These Th cell differentiations and cytokine productions are regulated by their lineage-specific master transcription factors, including T-bet/signal transducer and activator of transcription (STAT)4 for Th1 and GATA binding protein 3 (GATA3)/STAT6 for Th2 (Zhu, 2018). In experimental animal models, STAT4 deficiency in mice mildly improved renal function, whereas STAT6 deficiency markedly aggravated tubular injury following renal ischemia-reperfusion injury (IRI). T cells from STAT6 knockout mice expressed increased IFN-γ, but reduced IL-4 (Yokota et al., 2003). In addition, IL-4-deficient mice, representing defective Th2 immune responses, were also showed to suffer from a significantly aggravated functional and histological damage after IRI, especially the impairment of tubular regeneration. While IL-12- or IFN-γ-deficient mice with defective Th1 responses were completely protected from IRI by upregulating the expression of HO-1 encoding cytoprotective proteins (Yokota et al., 2003; Marques et al., 2006; de Paiva et al., 2009). A clinical study on biomarkers of AKI after cardiac surgery showed that Th1-induced IFN-γ and Th2-induced IL-4 and IL-13 increased after surgery associated with postoperative AKI (Moledina et al., 2019). The chemokines attracting leukocytes are generated from all types of intrinsic renal cells, such as endothelial, mesangial, tubular epithelial, interstitial cells, and podocyte, and regulate all the steps of leukocyte recruitments, including activation, adhesion, chemoattraction, and transmigration. In normal kidneys, the production of chemokines for proinflammatory T cells is very low but sharply increased under pathophysiological circumstances (Segerer et al., 2000; Chung and Lan, 2011). C-XC motif chemokine ligand (CXCL) 9 and CXCL10 are the two ligands of C motif chemokine receptor (CXCR) 3, which are mainly expressed on activated Th1 cells. The levels of CXCL9 and CXCL10 expression were elevated over time after IR to 72 h consistent with the loss of renal function (Fiorina et al., 2006). These data demonstrate Th1 cells are pathogenic for AKI, while Th2 cells are anti-inflammatory and protective.

Despite the protective role of Th2 in AKI, the benefit does not extend to the subsequent CKD. In 2012, the first direct evidence was provided that Th2 cells depraved renal fibrosis in the UUO mouse model. Thus, researchers put forward inhibition of Th2 differentiation from CD4^+^ T cells as a potential therapeutic intervention for renal fibrosis (Liu et al., 2012). Braga et al. demonstrated the absence of IL-4 was associated with alleviated UUO-induced renal fibrosis with better renal function that was contrary to its role in AKI. In the past, Th2 immunity was considered only as a simple regulator suppressing Th1 immunity to exert the anti-inflammatory function. Currently, the dual effects of Th2 become clear that these cell populations not only engage in protective events in reducing tissue inflammation and activating tissue repair, but also contribute to the development of tissue fibrosis when Th2 cytokine-mediated recovery processes become long-term, excessive, or dysregulated (Gieseck and Wynn, 2018). Furthermore, the study indicated that Th cell mediated renal injury by regulating macrophage differentiation into anti-inflammatory M2 and fibroblast collagen deposition rather than directly regulate the fibrotic response, and modulation of Th1:Th2 balance may be a potential strategy against renal fibrosis (Braga et al., 2012). These studies suggest that Th2-related immune responses exacerbate renal fibrosis contrary to the effect on AKI.

### T Helper 17

The third major Th cell population, Th17 cell, was discovered just several decades ago, which is controlled by the master transcription factors retinoic acid receptor-related orphan receptor (ROR)γt and STAT3 (Zhu, 2018). Th17 cells are generally perceived as detrimental factors for the pathogenesis of renal autoimmune diseases (Krebs et al., 2017; Dolff et al., 2019). Moreover, recent studies confirmed that Th17 cells also made a vicious influence on non-autoimmune AKI and CKD. Exposure to high-salt diets accelerated the transition from AKI to CKD for mice mediated by lymphocyte activities. Activated T cells, mostly positive for interleukin-17 (IL-17), were increased in the kidney after AKI and elevated with salt dietary intervention. The enhanced Th17 response hastened CKD and interstitial fibrosis and was inhibited by angiotensin II type-1 receptor (AT1R) antagonist, Losartan (Mehrotra et al., 2015). These studies demonstrate that Th17 cells play a pathogenic role in AKI and CKD mainly by IL-17 production.

The proinflammatory function of Th17 cells was achieved partly through IL-17 family of cytokines, which induced the mobilization and activation of neutrophils or participated in macrophage-mediated tissue injury (Kitching and Holdsworth, 2011; Mi et al., 2011; Cortvrindt et al., 2017). The effect of IL-17 on neutrophil recruitment was conducted in the acute phase of injury. IL-17 also contributed to chronic inflammation in lung (Wilson et al., 2010; Mehrotra et al., 2018) and liver (Tan et al., 2013) injuries in spite of low-frequency IL-17-producing cells in the later stage of inflammation, which was a dynamic multistep process (Miossec and Kolls, 2012; Tan et al., 2013). In renal obstruction models, IL-17A, a major member of the IL-17 cytokine family, facilitated renal fibrosis by RANTES-mediated leukocyte infiltration (Peng et al., 2015). However, the effects of therapy targeting IL-17 are contradictory. Several studies showed that systemic inhibition of IL-17 by antagonists significantly reduced renal fibrosis and neutrophil infiltration as well as pulmonary fibrosis and inflammation (Wilson et al., 2010; Miossec and Kolls, 2012; Mehrotra et al., 2017; Orejudo et al., 2019). Nevertheless, some other studies raised doubts due to blockade or deficiency of IL-17A having no beneficial effect on preventing renal fibrosis progression following severe IRI. The different severity of IRI might account for the contradictory conclusions (Thorenz et al., 2017; Rosendahl et al., 2019). Thus, whether the inhibitors of IL-17 cytokine family can be clinically applicated remains to be explored. And researchers need to focus on the confirmation of the specific subtype of cytokines in IL-17 family such as IL-17A and IL-17F associated with the pathogenic process of AKI and CKD. In addition, the severity and stages of renal injury are also worthy of consideration.

## Regulatory T Cells

Isolation and transfer of T lymphocytes infiltrated in kidney into T cell-deficient mice with renal IRI reduced functional and histological injury, thus, suggesting there may be a kind of renoprotective T-cell populations, which was confirmed as Tregs (Ascon et al., 2006). Foxp3 is currently the best marker to identify Tregs, which constitute 5–10% of the total CD4^+^ T-cell populations. Despite the relative low frequency, Tregs are regarded as crucial orchestrators of the regulation of inflammation, the maintenance of immune tolerance, and homeostasis (D'Alessio et al., 2019). In animal experiments, there was significant recruitment of Tregs into kidneys 3- and 10-days postischemia. These infiltrated Tregs promoted tubular proliferation and reduced proinflammatory cytokine generation, and depletion of Tregs worsened renal function and mortality (Gandolfo et al., 2009). C-C motif chemokine ligand (CCL20), expressed by tubular, endothelial, and interstitial cells, is a key attractor for the influx of Tregs as well as Th17 into injured kidneys. CCL20 was upregulated in three models of renal injury induced by over-dose folic acid (FA), cisplatin, and UUO. In FA-induced AKI, the strong upregulation of CCL20 was confirmed at day 2 of renal injury and persisted for 7 days. CCL20 was increased in human kidneys and urine with AKI and urinary CCL20 was associated with severity (Gonzalez-Guerrero et al., 2018). In one clinical study of sepsis-associated AKI, the ratio of Tregs in peripheral blood might provide a potential biomarker to accurately evaluate the prognosis of sepsis (Chen et al., 2015). Another research on patients with AKI demonstrated the positive effect of T-cell immunoglobulin and mucin domain 3 (TIM-3) on Treg protective function (Dong et al., 2019). The mechanisms of Treg protective role in kidney injury refer to the secretions of immunosuppressive cytokines and pro-repair mediators. IL-10 is the major anti-inflammatory molecule produced by Tregs (D'Alessio et al., 2019). IRI induced a significant increase in IL-10^+^ Tregs in the repair phase (Kinsey et al., 2010). Treg reduction caused the increase of neutrophil and macrophage infusion and innate cytokine transcription. Furthermore, transfer of wild-type Tregs into immunodeficient mice prevented renal IRI, but transfer of IL-10-deficient Tregs did not. These data indicate that Tregs regulate renal IRI in early injury stage by suppression of the innate immune responses in an IL-10-mediated manner (Kinsey et al., 2009). In addition, Treg-derived adenosine activates adenosine 2A receptor (A_2A_R) is expressed on immune cells suppressing inflammation and improving renal function decline after IRI through a programmed cell death (PD-1)-dependent mechanism, which is regulated by CD73, the final enzyme participating in the extracellular adenosine production (Kinsey et al., 2012). Endogenous Toll-like receptor 9 (TLR9) is also an important regulator of AKI by promoting Treg recruitment (Alikhan et al., 2016). These studies imply that expansion of Tregs might be a potential strategy for preventing AKI.

*In vitro*, Tregs modulated macrophages by inhibiting their activation and downregulating the effector phenotype of macrophages, leading to alleviate chronic kidney injury. The Treg-macrophage inhibitory interaction was transforming growth factor-β (TGF-β)-dependent (Mahajan et al., 2006). Besides that, CD226 deficiency on Tregs exacerbated renal fibrosis in the UUO model by upregulating Th2-related cytokines like IL-4 (Mu et al., 2020). Recently, a single-cell RNA sequencing study showed that tissue-resident IL-33R^+^ and IL-2Ra^+^ Tregs markedly increased following injury in the two mouse models of either kidney repair or fibrosis. Mice with expansion of this population before injury were protected from renal injury and fibrosis. However, despite Tregs showing a upregulation of regenerative and proangiogenic pathways in the repair phase, they expressed markers related to hyperactivation and fibrosis in the fibrotic environment, suggesting the plasticity in Treg function (do Valle Duraes et al., 2020). Many interventions targeting Treg expansion were confirmed to attenuate kidney injury, including the CCL20 blocking agent (Zuk and Bonventre, 2016), the IL-2/anti-IL-2 complex (IL-2C) (Kim et al., 2013), IL-233(Stremska et al., 2017), resolvin D1 (Luan et al., 2020), mesenchymal stem cell–derived extracellular vesicles (Song et al., 2020), and oxidized ATP (oATP) (Koo et al., 2017). These data suggest that it is necessary to understand the plasticity and heterogeneity of Tregs and figure out the precise function of each Treg subset.

### CD8^+^ T Cells

After IRI, renal IFN-γ-producing CD8^+^ T cells were increased (Ascon et al., 2008). Germ-free mice encountered more severe IRI, which was associated with the enhancement of CD8^+^ T cell trafficking to the kidney (Jang et al., 2009). However, unlike CD4^+^ T cells, CD8^+^ T cell deficiency did not alter the renal outcomes of IRI (Burne et al., 2001). In spite of this, the detailed roles of CD8^+^ T cells on AKI are yet to be completely determined.

In the UUO mouse model of renal fibrosis, genetic ablation of CD8^+^ T cells increased renal interstitial fibrosis by promoting BM-derived monocyte-to-fibroblast transition, whereas, adaptive transfer of CD8^+^ T cells to CD8 knockout mice decreased fibrosis, which indicated that CD8^+^ T cells might have an anti-fibrotic effect on kidneys. A further study discovered that depletion of CD8^+^ T cells resulted in the higher expression of IL-4 and GATA3 and lower expression of IFN-γ and T-bet on CD4^+^ T cells, suggesting that CD8 knockout primed the immune response of Th1 skewing to that of Th2 (Dong et al., 2016). The infiltrated CD8^+^ T cells from obstructed kidneys expressed perforin, granzyme, and Fas ligand (FasL) that were related to cytotoxicity. The activation of CD8^+^ T cells required the inflammatory milieu, in which chemokines like CCL2, CCL3, CCL4, and CCL5 existed obviously. In addition, CD8^+^ T cells were distributed around fibroblasts to mediate the apoptosis of these profibrotic cells. Moreover, CD11c expression made CD8^+^ T cells express higher levels of the cytotoxicity-associated genes, and *in vitro*, CD11c^+^ CD8^+^ T cells induced fibroblast death (Wang et al., 2016). Thus, promoting CD8^+^ T cell recruitments may be an effective mechanism, by which clusterin (Guo et al., 2016) and astaxanthin (Diao et al., 2019) protect against renal fibrosis.

### Double-Negative T Cells

*Double-negative T* cells identified by CD4- and CD8- are an unconventional component of αβT cells, which constitute 20–38% of the αβT cell pool in normal kidneys of mice. In contrast, the frequency levels in the lymph nodes and spleen are lower in only 5–10%. In a steady state, DNT cells in the kidney display an activated phenotype expressing less CD62L with higher expressions of CD44 and CD69 compared to CD4^+^ and CD8^+^ counterparts. Besides that, kidney-resident DNT cells proliferate actively under the steady state and suppress CD4^+^ T cell proliferation *in vitro* (Martina et al., 2016). Many previous studies showed that DNT cells producing IL-17 and IFN-γ expanded in patients with systemic lupus erythematosus (SLE) (Crispin et al., 2008) and were a potential biomarker for SLE (Alexander et al., 2020). They also contributed to other autoimmune diseases such as type-1 diabetes (Ford et al., 2007), Sjögren's syndrome, and psoriasis (Brandt and Hedrich, 2018). In response to IR-induced AKI, DNT cells expanded significantly and become the dominant subsets of the early responders. IL-10 and IL-27 cytokines were markedly expressed in DNT cells in a steady state but altered in IRI with significantly increased IL-10 and slight decreased IL-27 that suggested that DNT cells might be beneficial to prevent AKI depending on IL-10 as conventional cytokine from Tregs. Further study confirmed this hypothesis by adaptive transfer experiments (Martina et al., 2016). DNT cells alleviated cisplatin-induced dysfunction and structure damage in AKI by reducing apoptosis in proximal tubular epithelial cells (PTECs) of the kidney (Gong et al., 2020). Another mechanism of DNT cells in the suppression of immune responses was directly killing effector T cells by antigen-specific recognition and Fas/FasL or perforin/granzyme pathway(Chen et al., 2004; Voelkl et al., 2011). In human kidneys, DNT cells also accounted for a high proportion of T cells, which suggested the prospect of DNT cells for clinical translation (Martina et al., 2016). These studies demonstrate that DNT cells protect mice from AKI in the early stage by regulating cytokine production and the cytotoxic effect, and their roles on CKD remain to be understood.

### Innate Lymphoid Cells

Innate lymphoid cells originate from common progenitors with T and B lymphocytes but lack adaptive antigen receptors, which are a heterogeneous population and the innate counterparts of T cells. Based on the major cytokines ILCs produce and the master transcription factors driving their differentiation, ILCs are distinguished from three groups: IFN-γ-producing T-bet^+^ ILC1s, GATA3^+^ ILC2s secreting IL-5, IL-9, IL-13, and amphiregulin, and RORγt^+^ ILC3s producing IL-22 and IL-17. ILC1s, ILC2s, and ILC3s correspond to Th1, Th2, and Th17, respectively (Vivier et al., 2018). ILCs are found predominantly resident in barrier organs like the gut, the lung, and the skin, where they maintain tissue homeostasis, regulate against infection and contribute to immune-mediated diseases in mice and humans (Gasteiger et al., 2015). Recently, a novel regulatory subpopulation of ILCs existing in the gut has been identified called ILCregs, which harbor a unique gene identity distinct from that of ILCs or Tregs. ILCregs secret IL-10 to activate ILC1s and ILC3s, causing protection against innate gut inflammation (Wang S. et al., 2017), but whether they can function as conventional Tregs for reno-protection remains unclear. These data suggest ILCs have a large family and the members exert different functions.

In healthy murine and human kidneys, ILC2s are the major ILC population and play a similar role in AKI and CKD to Th2 due to their co-participating type 2 immunity (Wang Y. M. et al., 2017; Gieseck and Wynn, 2018). Given the protective role of Th2 and type 2 immunity on AKI, the intent of the researchers is to find whether ILC2s are also beneficial to kidney injury. Expanding ILC2s by IL-25 or IL-33 released from damaged epithelial cells significantly improved renal function and alleviated injury after IR with greater amounts of Th2 cytokines such as IL-4, IL-5, and IL-13, which induced M2 macrophage subtype and suppressed M1 *in vitro*. Furthermore, adaptive transfer of ILC2s identically reduced renal functional and histological damage as well as enhancing M2 induction and amphiregulin production in the kidney (Huang et al., 2015; Cao et al., 2018; Gieseck and Wynn, 2018). But the concrete effects of IL-33 treatment depended on the dosage, the duration, and the injury type, which might reverse the advantageous role to a deleterious one (Cameron et al., 2019a). IL-233, a novel hybrid cytokine bearing the activities of IL-2 and IL-33, increased the frequency of ST2-bearing ILC2s in both blood and kidneys to protect mice from IRI (Stremska et al., 2017). In a doxorubicin-induced nephrotoxic renal injury model, IL-233 treatment not only augmented anti-inflammatory cytokines and attenuated proinflammatory cytokine thus reducing renal inflammation, injury, and fibrosis, but also promoted regeneration with increased expression of genes related to renal progenitor cells and nephron segments (Sabapathy et al., 2019). However, a loss of ILC2s does not alter the severity of IR-induced renal injury suggesting the redundancy of ILC2s for IRI protection, which may be due to the compensation of another type 2 immune cell activation (Cameron et al., 2019b).

Expanded ILC2s by IL-33 were also confirmed to ameliorate glomerulosclerosis in mice with Adriamycin-induced CKD (Wang et al., 2015). Nevertheless, the anti-fibrotic role of ILC2s needs to be well-argued since type 2 immunity is commonly considered as a profibrotic factor such as Th2 response mentioned above (Gieseck and Wynn, 2018). In summary, ILCs alleviate AKI by mechanisms similar to Th2, and their effects on CKD remain to be explored.

### Innate-Like T Lymphocytes

Innate-like T lymphocytes, including NKT cells, γδT cells, and MAIT cells, are a significant component of innate immunity and they along with ILCs hold a unique capacity for innate responses to maintain homeostasis of the gut (Constantinides, 2018), and the lung (Borger et al., 2019). Their early responses to renal injury also deserve special attention.

### Natural Killer T Cells

Natural killer T cells are an unusual T lymphocyte subpopulation that co-expresses the natural killer receptors and TCRs, serving as a bridge between innate and adaptive immunity. These cells are lipid-sensing innate-like T cells, which express semi-invariant αβTCR only recognizing glycolipid antigens presented by CD1d, a major histocompatibility complex (MHC)-I-like molecular (Sun et al., 2019). According to the Vα14-Jα18 in TCRs, NKT cells are categorized into type I and type II NKT cells. The former type, also named iNKT cells, is sensitive to glycolipid α-galactosyl ceramide (αGalCer) and the latter is activated by the self-glycolipid 3-sulfated β-galactosyl ceramide (sulfatide) (Jahng et al., 2004).

Natural killer T cells are not sensitive to non-protein DAMP molecules, but alarms like IL-33 cytokines that are tissue-derived nuclear proteins released after damage, activate NKT cells to recruit neutrophils by IFN-γ and IL-17A release (Ferhat et al., 2018). By this means, NKT cells regulate the initial process of sterile inflammation in the kidney (Li et al., 2007). IFN-γ cytokines are the important downstream effector molecular of NKT cells. Spontaneous, local, and probably extravascular activation events of NKT cells in the liver and kidney result in the local secretion of IFN-γ (Zeng and Howard, 2010; Aguiar et al., 2015). NKT cells injured kidney vascular endothelial cells by perforin-mediated pathway and tubular epithelial cells by tumor necrosis factor **(**TNF)-α/FasL pathway, leading to AKI with hematuria in mice. The human CD56^+^ T cells, a counterpart of mouse NKT cells, exerted their similar functions (Uchida et al., 2018). Natural Immunoglobulin M (IgM) anti-leukocyte autoantibodies (IgM-ALAs) (Lobo et al., 2017) and A2AR agonists (Li et al., 2012) attenuated AKI by suppressing NKT cells. However, studies on type II NKT cells found that they abrogated kidney IRI. *In vitro*, type II NKT cells attenuated tubular apoptosis after transient hypoxia through hypoxia-inducible factor (HIF)-1α and IL-10 pathways (Yang et al., 2011).

In adenine-induced renal injury, administration of αGalCer to activate iNKT cells reduced renal fibrosis. CD1d-dependent NKT cells improved non-alcoholic fatty liver disease (NAFLD)-associated CKD *via* reducing renal inflammation, mesangial cell proliferation, and tubular cell apoptosis (Alhasson et al., 2016). Besides that, IL-22 cytokines produced by NKT cells have been proved to play a protective or pathogenic role in chronic inflammation depending on the nature of the influenced tissues and the cytokine microenvironment (Witte et al., 2010; Dudakov et al., 2015). These data imply that NKT cells in the kidney have dual effects depending on the polytropic inflammatory milieu.

### Gamma Delta T Cells

Gamma delta T cells are another type of T cells, distinguishing from αβT cells based on the distinct TCR types. They are triggered by stress-induced ligands from aberrant cells without antigen processing and presentation, thus acting in the initial phase of injury and playing a major role in bridging innate and adaptive immune responses (Patil et al., 2015).

Gamma delta T cells are one of the sources of IL-17A that is increased in cisplatin-induced AKI and UUO-induced renal fibrosis (Huen and Cantley, 2017; D'Alessio et al., 2019). However, depletion of γδT cells did not ameliorate cisplatin-induced renal injury, indicating γδT cells were unnecessary to injury (Chan et al., 2014). Even so, γδT cells responded very quickly to human AKI. The decrease in circulating γδT cells and increase in kidney γδT cells demonstrated the possibility of their migration from circulation to kidney tissues. The changes of infiltrated γδT cells were correlated with the elevated biomarkers of tubular stress or injury (Gocze et al., 2018). Analysis of kidney biopsy tissues from patients with renal fibrosis showed that larger numbers of γδT cells were infiltrated in kidneys with lower estimated glomerular filtration rate (eGFR), which suggested the negative correlation between γδT cell number and loss of renal function. In addition, fibrotic tissues contained significantly more Vδ1^+^ γδT cells, and CD161^+^ γδT cells displayed an innate-like cytotoxic phenotype. Furthermore, the localization and the expression of IL-17A of γδT cells implied that they might mediate the survival of PTECs (Law et al., 2019a). These data from animal experiments and clinical studies showed that γδT cells are pathogenic factors for renal injury and fibrosis by promoting IL-17A production.

### Mucosa-Associated Invariant T Cells

Mucosa-associated invariant T cells are one of the innate-like T cell subtypes recognizing riboflavin metabolites depending on the MHC-related molecule 1. When activated, they rapidly produce various proinflammatory cytokines suggesting their detrimental roles in chronic inflammatory diseases (Howson et al., 2015). MAIT cells are enriched in the gut, liver, and lung to defend against pathogen attack (Le Bourhis et al., 2010). In recent years, only one article published in Journal of the American Society of Nephrology (JASN) addressed that tissue-resident MAIT cells in human kidneys might contribute to the fibrotic process of CKD by complex interactions with PTECs. In this study, researchers found that MAIT cells were increased in the tissue samples from fibrotic kidneys compared with those from non-fibrotic kidneys. And the numbers of MAIT cells were correlated with loss of kidney function. Furthermore, these MAIT cells accumulated adjacent to PTECs, and highly expressed activation marker CD69 cytotoxic molecules perforin and granzyme B, which led to PTEC necrosis (Law et al., 2019b). Despite that, further studies of MAIT cells are necessary to unequivocally determine the non-redundant role in CKD pathogenesis.

Recently, the application of high-dimensional cellular analyses, such as single-cell RNA sequencing (scRNA-seq), has helped researchers to figure out detailed characterization of immune cell population in kidney disease. The research on patients with kidney transplant rejection showed that recipient-origin T-cells expressed proinflammatory genes, whereas donor-origin T-cells expressed oxidative phosphorylation genes, which indicated that T cells from two sources had distinct transcriptional profiles (Malone et al., 2020). In addition, the technique of scRNA-seq has been used to identify CD4^+^ tissue-resident memory T cells with a Th17 signature in the kidney of patients with antineutrophil cytoplasmic antibody–associated glomerulonephritis and dysfunction of CD8^+^ T cells in patients with SLE and IgA nephropathy (Krebs et al., 2020; Maria and Davidson, 2020). However, this advanced technique is rarely used in studies on AKI, which may be due to the lack of samples from patients of AKI. Our team has recently identified four subcluster macrophages at different time points in the I/R model and analyzed the transition of Arg1^+^ macrophages (Cluster 1) into Ccr2^+^ macrophages (inflammatory Cluster 0) (Zhu et al., 2021). In a further study, we will focus on the identification of lymphocyte characteristics and analyze different functions of subtypes in AKI and CKD.

## Versatile Lymphocytes in AKI and CKD: Chronological Responses and Plasticity

**Figure 2:**
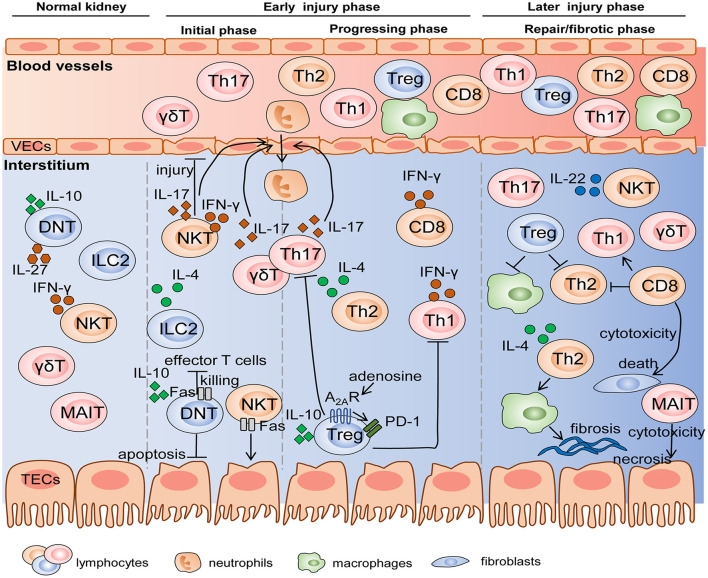
The mechanisms of diverse lymphocyte subsets on AKI and renal fibrosis in the different stages. Innate and innate-like lymphocytes reside in normal kidneys and become early responders to renal injury. Subsequently, differentiated adaptive T cells migrate into the kidney in progressing phase, and some of these cells like Treg and MAIT participate in the repair or fibrotic process. Cell-cell interactions are achieved by the production of various cytokines and cytotoxicity mediated by FasL/Fas. VEC, vascular endothelial cell; TEC, tubular epithelial cell.

The pathogenesis of AKI and CKD is a complicated process with the involvement of multicellular and multifactor in the above elaboration. These pleiotropic immune cells respond to injury in a chronological order according to their immunological characteristics and mechanisms of damage responses (shown in Figure 2). It is indispensable to determine the “time window” of distinct lymphocytes, which will guide precise therapies targeting corresponding effector cells at different stages of injury.

In general, innate and innate-like lymphocytes respond rapidly to organ injury. For instance, NKT cells and γδT cells were immediate participants in the hyperacute immune responses following the first 6 h of trauma and hemorrhagic shock (Manson et al., 2019). In the kidney, Hochegger et al. confirmed that αβT cells were the major effector cells, whereas γδT cells acted as mediator cells in the first 72 h of renal IR-induced injury, bridging immediate innate and subsequent adaptive immunity. Deficiency of αβT cells reduced renal functional and histological damage 72 h after IR, but had no influence on renal function 24 h after IR. Moreover, infiltration of γδT cells into kidney was equal in αβT cell-deficient mice and wild-type mice, whereas, lack of γδT cells led to the significant decrease in αβT cells until 120 h after IR, suggesting an interaction between αβT cells and γδT cells. These data indicated that γδT cells functioning in the early injury stage might be a driver of CD4^+^ and CD8^+^ T responses in the later stage (Hochegger et al., 2007). DNT cells are also early responders, which significantly expanded, and rapidly became the major subpopulation 3 h after IRI and remained dominant until 24 h. However, they decreased to the level below steady state by 72 h after IRI. DNT cells are a dominant kidney-resident subset and largely produce cytokines at the steady state, which may be one explanation why they respond so quickly to AKI in an innate-like manner (Martina et al., 2016). Besides that, NKT cells and ILC2s are also considered as initial responders to kidney injury due to their innate-immune and tissue-resident properties. They are activated and expanded by molecules released from damaged cells without the involvement of antigen-presenting cells, while adaptive T cells need to go through the process of differentiation, thus determining their delayed responses to kidney injury (Cao et al., 2018; Ferhat et al., 2018). Therefore, we cannot ignore the crucial effects of innate and innate-like lymphocytes, a pioneer of AKI, whether they are beneficial or detrimental.

After innate immunity activation, adaptive T lymphocytes come on stage and they persist activated from acute to chronic phase. Th1/Th2 balance regulated renal injury 24 and 48 h after IR (Marques et al., 2006). Th17 cells produced IL-17 cytokines to recruit neutrophils, thus influencing early kidney injury events with dramatical enhancement at day 1 and day 3 post IR and recovery to the baseline at day 7. The second expansion of Th17 cells existed with the high-salt diet hit (Mehrotra et al., 2015, 2019). Tregs trafficked into the kidney to suppress the function of Th1, Th2, and Th17 at day 3 post IR and involved in repair at day 10 (Gandolfo et al., 2009). In addition, Th2 also mediated tubular reparation and promoted fibrosis with excessive repair (Marques et al., 2006). In summary, early responses of innate and resident lymphocytes along with later responses of adaptive T cells participate in the pathogenesis of AKI and T cells related to tissue repair or fibrosis engage in the subsequent CKD progression.

In renal IRI, a clear M1-to-M2 phenotypic switch occurs, which provides the possibility that the same cell possesses plasticity under the changes of microenvironment (Tang et al., 2019). Like macrophages, the plasticity of CD4^+^ T cells is also reflected in immune-mediated kidney disease (Krebs and Panzer, 2018). The expressions of their lineage-specific master transcription factors are dynamic and cross-regulation. The co-expressions of these transcription factors result in CD4^+^ T cell plasticity (Zhu, 2018). Researchers found the conversion of Tregs to IL-17^+^ phenotypic cells enhanced renal fibrosis in UUO mice, which was prevented by inhibition of histone deacetylase (HDAC) activity suggesting the importance of epigenetic modifications (Wu et al., 2017). Besides CD4^+^ T cells, ILC2s can also switch into ILC1s or ILC3s due to yet unknown triggers (Krabbendam et al., 2018). Differentiated T cells have the potential to dedifferentiate and undergo the shift to another subtype, which is driven by a complex network of signals under the microenvironment of AKI and CKD. Nevertheless, whether this plasticity occurs, which form it presents with and which signals trigger it remains to be further studied, aiming to provide enough evidence of the possibility of modulating cell-cell plasticity to alleviate inflammatory kidney diseases (Rajendran et al., 2019).

## Potential Therapies Targeting Lymphocytes of AKI and CKD

Based on the versatile lymphocytes engaging in the process of AKI and CKD, what targeting these players may be potential strategies for ameliorating the inflammation-mediated diseases, including the interventions of lymphocyte activities *in vivo*, the adaptive transfers of ex-expanded beneficial lymphocytes like Tregs, and the treatments based on the gut-kidney axis.

### Targeting of Lymphocytes *in vivo*

BX471, a CCR1 blocker, reduced renal fibrosis in mice of UUO by decreasing the T cell infiltration, which represented that blocking chemokines and respective receptors might be a potential therapeutic strategy for alleviating renal fibrosis (Anders et al., 2002). However, CCL20 blockade increased the severity of FA-induced AKI, which might be related to a lower infiltration of Tregs in spite of a decrease in proinflammatory Th17 influx (Gonzalez-Guerrero et al., 2018). Therefore, the treatments targeting chemokines and their receptors should be considered for their non-specificity for recruiting lymphocytes.

Inhibiting IL-18 by IL-18 binding protein (Bp) also impaired T cell infiltration and reduced IR-induced renal fibrosis (Liang et al., 2018). In addition, IL-25 treatment protected mice against IRI by elevating ILC2 frequency (Huang et al., 2015). Interestingly, the administration of IL-33 is a double-edged sword. On the one hand, IL-33 ameliorated IR-induced AKI by expanding ILC2 and Tregs (Cameron et al., 2019a). On the other hand, soluble ST2 (sST2), a decoy receptor that neutralizes IL-33 activity, also reduced renal IRI by lowering CD3^+^ cell level (Liang et al., 2017). The dosage of IL-33, the duration of administration, and the severity and the stage of renal injury may remarkably alter the responses performed to be beneficial or deleterious. IL-233 is a hybrid cytokine bearing IL-2 and IL-33. IL-233 increased the number of Tregs and ILC2s in doxorubicin-induced nephrotoxic renal injury and enhanced Treg influx in IRI, thus playing a protective role in AKI of the two models (Stremska et al., 2017; Sabapathy et al., 2019). Moreover, IL-17Rc inhibited Th17 activation leading to significantly mitigate renal fibrosis (Mehrotra et al., 2017). The administration of an IL-36R antagonist after UUO attenuated tubulointerstitial lesions (TILs) that might be associated with the reduction of Th17 differentiation (Chi et al., 2017). IL-2/anti-IL-2 complex (IL-2C) protected against mouse IRI by inducing Treg expansion (Kim et al., 2013). Thus, various cytokines as the main effector molecules are the valuable target of T cells to treat AKI and CKD.

Besides that, as one of the inhibitors of delayed rectifier K^+^-channel, Kv1.3 like margatoxin (Abe et al., 2019) and YM58343/BTP2 (Mehrotra et al., 2019) were expressed on T lymphocytes, and also found to acute injury and chronic fibrosis in rat kidney. T cell infiltration was decreased by a wingless-type (Wnt)/β-catenin pathway inhibitor, ICG-001, which therefore prevented CKD in a 5/6 nephrectomy model (Xiao et al., 2019). Many other agents, including losartan (Mehrotra et al., 2015), periodate-oATP (Koo et al., 2017), rapamycin (Chen et al., 2016), and resolvin D1 (Luan et al., 2020) augmented Treg accumulation to reduce renal damage and promote recovery. Mesenchymal stem cells (MSCs) supplemented with a vitamin D receptor agonist inhibited Th17 differentiation (Duffy et al., 2014). Astaxanthin promoted CD8^+^ T cell recruitment (Diao et al., 2019), and a HDAC inhibitor, trichostatin A (TSA), lowered CD4^+^ IL-17^+^ T cell percentage (Wu et al., 2017). All of these treatments prevented renal fibrosis. Besides that, Kynorenin as an important immune modulator was found highly predictive for “major adverse kidney events” of contrast induced AKI (Reichetzeder et al., 2017). These data suggest how to expand beneficial T cells and reduce pathogenic ones *in vivo* is the focus issue. In summary, the treatments targeting lymphocytes *in vivo* were listed in Table 3.

**Table 3:** The treatments targeting of lymphocytes *in vivo*.

**Agents**	**Descriptions**	**Species**	**Models**	***In vivo* effects**	**References**
IL-18 Bp	A IL-18 inhibitor	Mouse	IR	T cell infiltration ↓ Renal fibrosis ↓	Wu et al., 2017
soluble ST2 (sST2)	A decoy receptor Neutralizing IL-33 activity	Mouse	IR	T cell infiltration ↓ Renal function ↓ Renal injury score ↓ Renal fibrosis ↓	Krabbendam et al., 2018
BX471	A CCR1 blocker	Mouse	UUO	T cell infiltration ↓ Renal fibrosis ↓ Histopathological injury ↓	Hochegger et al., 2007
Margatoxin	A Kv1.3-channel inhibitor	Rat	UUO	T cell infiltration, proliferation and cytokine production ↓ Renal fibrosis ↓	Rajendran et al., 2019
ICG-001	A Wnt/β-catenin pathway inhibitor	Rat	5/6 nephrectomy	T cell infiltration ↓ Renal function ↓ Renal fibrosis ↓	Anders et al., 2002
YM58343/BTP2	A calcium-channel inhibitor	Rat	IR	Th17 activation ↓ Renal function ↓ Acute renal injury ↓ Renal fibrosis ↓	Mehrotra et al., 2019
IL-17Rc	The IL-17Rc soluble receptor	Mouse	IR	Th17 activation **↓** Renal fibrosis **↓**	Mehrotra et al., 2017
Losartan	An angiotensin receptor 1 (AT1) antagonist	Mouse	UUO	Treg accumulation **↑**	Mehrotra et al., 2015
		Rat	IR	Th17 number ↓ Renal function ↓ Renal fibrosis ↓	
IL-36Rn	IL-36R Antagonist	Mouse	UUO	Th17 differentiation ↓ Renal fibrosis ↓	Chi et al., 2017
MSCs and paricalcitol	Co-administration of MSCs and a vitamin D receptor agonist	Mouse	UUO	CD4+ and CD8+ T cell accumulation ↓	Duffy et al., 2014
				Th17 differentiation ↓ Renal fibrosis ↓	
Trichostatin A (TSA)	A histone deacetylase (HDAC) inhibitor	Mouse	UUO	CD4+IL-17+ T cell percentage ↓ Renal fibrosis ↓	Maria and Davidson, 2020
Periodate-oxidized ATP (oATP)	A P2X7 receptor (P2X7R) antagonist	Mouse	IR	Treg infiltration **↑** Tubular injury ↓ Renal fibrosis ↓	Koo et al., 2017
IL-2/	–	Mouse	IR	Treg number **↑** Renal function **↑** Tubular injury ↓ Renal fibrosis ↓	Kim et al., 2013
Anti-IL-2 complex (IL-2C)					
Rapamycin	A mTOR inhibitor	Mouse	IR	Treg number **↑** Acute kidney injury ↓ Renal fibrosis ↓	Liang et al., 2018
Resolvin D1	An endogenous lipid mediator	Mouse	IR	Treg percentage **↑** Tubular injury ↓	Luan et al., 2020
IL-233	A hybrid cytokine bearing IL-2 and IL-33	Mouse	Cisplatin- and doxorubicin-induced nephrotoxic renal injury	Treg and ILC2 number and proportion **↑** Renal function **↑** Tubular injury ↓ Renal fibrosis ↓	Sabapathy et al., 2019
			IR	Treg number **↑** Renal function **↑** Tubular injury ↓	Stremska et al., 2017
Astaxanthin	A natural and nontoxic xanthophyll carotenoid	Mouse	UUO	CD8+ T cell recruitment **↑** Renal fibrosis ↓	Diao et al., 2019
IL-25	–	Mouse	IR	ILC2 frequency **↑** Tubular injury ↓	Huang et al., 2015
IL-33	–	Mouse	IR	ILC2 and Treg number **↑** Renal function **↑** Tubular injury ↓	Cao et al., 2018

### Adaptive Transfer of Ex-expanded Lymphocytes

In animal experiments, adaptive transfer of beneficial lymphocytes such as Tregs, DNT cells, and ILC2s can effectively reduce renal injury caused by IR and nephrotoxins. Treg-based cellular therapy has been extensively studied in recent years. Studies have shown that Tregs protect allografts in kidney transplant models of mice due to their immunosuppressive function. However, clinical translation is challenging. Treg preparations, dose, and frequency are worthy of consideration. In addition, the instability and variability of Tregs arise safety concerns, thus Tregs should be identified and quantified prior to transplant trials (Chandran et al., 2017; Tang and Vincenti, 2017; Zwang and Leventhal, 2017; Savage et al., 2018). Nevertheless, Treg therapy has not been applied to treat patients with AKI and CKD. The efficacy and safety need to be further explored. The modifications of beneficial lymphocytes *in vitro* regulate their protective effects. CD73-deficient or A_2A_R-deficient Tregs failed to protect mice from IRI suggesting that the response to adenosine was required to Treg to suppress inflammation, and the induction of A_2A_R on Tregs augmenting the protective functions verified this hypothesis (Kinsey et al., 2012). TLR9 expression regulated the migration of transferred Tregs into the kidney (Alikhan et al., 2016). IL-233-treated mice were preferred donors, of which Tregs exerted a beneficial effect on alleviating renal IRI, and this protection depended on the cell dosages (Stremska et al., 2017). Ex-expanded Tregs also promoted renal repair and rapamycin, an inhibitor of mammalian target of rapamycin (mTOR), enhanced their pro-repair effects (Gandolfo et al., 2009; Chen et al., 2016). Besides that, DNT cells protected mice against both cisplatin- and IR-induced renal injury, and the process of the latter was in an IL-10-dependent manner (Martina et al., 2016; Gong et al., 2020). For ILC2s, IL-33 and IL-233 treatment promoted their protection of the kidney against IRI. Furthermore, human-derived ILC2s ameliorated renal damage in mice (Stremska et al., 2017; Sabapathy et al., 2019). In conclusion, the administration of ex-expanded beneficial lymphocytes might prevent renal injury, which is listed in Table 4.

**Table 4:** Adaptive transfer of ex-expanded lymphocytes.

**Subsets**	**Dosages**	**Species of recipients**	**Methods**	**Effects**	**References**
Tregs	1.0 × 10^5^ cells	Mouse	Before IR	Wild-type Tregs protected mice from IRI.	Dong et al., 2019
				CD73-deficient or A_2A_R-deficient Tregs failed to protected mice from IRI.	
				Pharmacologic stimulation of A_2A_R on Tregs augmented the protective functions.	
Tregs	2.0 × 10^6^ cells	Mouse	24 h before cisplatin administration	Adaptive transfer of wild-type Tregs resulted in less severe cisplatin-induced AKI than that of TLR9-deficient Tregs.	Kinsey et al., 2010
				TLR9 promoted Treg recruitment.	
Tregs	50 × 10^3^ cells	Mouse	24 h before IR	Tregs from IL-233-treated mice played better protective roles in IRI at lower doses (50 × 10^3^).	Mu et al., 2020
	100 × 10^3^ cells			Tregs at higher doses (100 × 10^3^) had no protective roles.	
Tregs	2.0 × 10^6^ cells	Mouse	24 h after IR	Rapamycin-treated Tregs enhanced beneficial effects on reducing IRI on the early (3 d) and later (14 d) repair stages.	Krabbendam et al., 2018
Tregs	1.2 × 10^6^ cells	Mouse	24 h after IR	Tregs promoted kidney repair after IRI.	Liu et al., 2018
DNT cells	1.0–1.5 × 10^6^ cells	Mouse	24 h before cisplatin administration	DNT cells attenuated cisplatin-induced AKI.	Alexander et al., 2020
DNT cells	2.5 × 10^6^ cells	Mouse	24 h before IR	DNT cells protected mice from AKI in a IL-10-dependent manner.	Diao et al., 2019
ILC2s	1.0 × 10^6^ cells	Mouse	24 h before IR	IL-33-treated ILC2s prevented renal injury in an Areg-dependent manner.	Wang Y. M. et al., 2017
				Human-derived ILC2s ameliorated renal IRI in mice.	
ILC2s	5.0 × 10^5^ cells	Mouse	24 h before IR	IL-233-treated ILC2s protected mice from IRI	Mu et al., 2020

### Potential Therapeutic Strategies Targeting T Cells Based on the Regulation of the Gut-Kidney Axis

Recently, the crosstalk of gut and kidney has been a hot spot. Gut-derived metabolites, short-chain fatty acids (SCFAs), were confirmed to prevent AKI. *In vitro*, SCFAs regulated the inflammatory process, in which dendritic cells were inhibited in the capacity of induced CD4^+^ and CD8^+^ T cell proliferation (Andrade-Oliveira et al., 2015). Acetate, one of the SCFAs, ameliorated AKI by redressing oxidant-antioxidant imbalance of T cells dependent on nicotinamide adenine dinucleotide phosphate (NADPH) oxidase (NOX2)/reactive oxygen species (ROS) signaling (Al-Harbi et al., 2018). The intestine microbiota has a potential immunoregulatory role in CKD. The pathogen overgrowth in the gut under the metabolic alterations of uremia, and an increase of bacteria or their component translocation may activate systemic inflammation concerned with lymphocyte-participated immunity (Anders et al., 2013). The development and application of multi-omics analysis technique have helped us to screen out specific-altered gut microbiota and metabolites that may be associated with T cell immunity modulation in kidneys.

**Figure 3:**
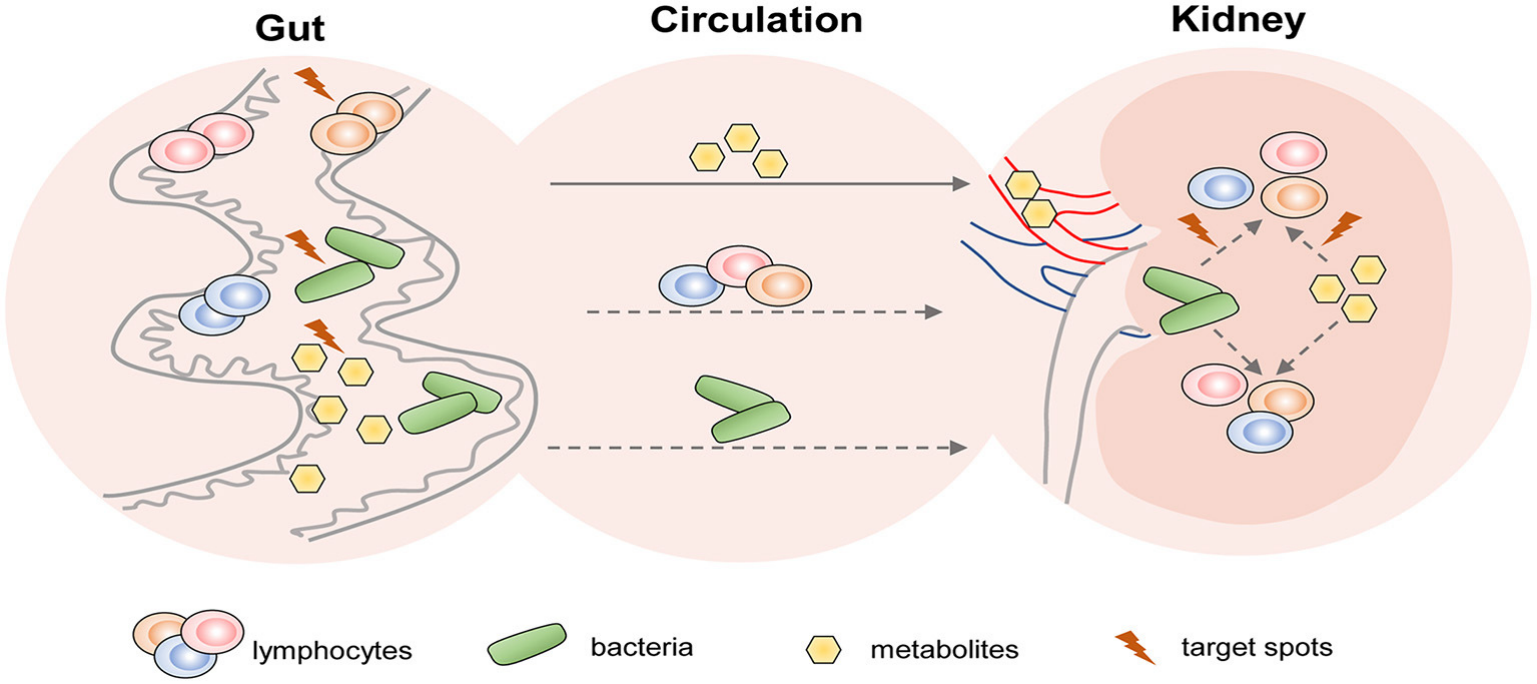
The potential therapeutic strategies targeting T cells of AKI and CKD based on the gut-kidney axis. Based on the theory of “gut-kidney axis,” gut dysbiosis and immune activation induced by kidney diseases including AKI and CKD, in turn, aggravate kidney damage. Thus, there are three aspects of potential therapeutic target spots in the gut to prevent kidney diseases: (1) modulation of gut microbiota; (2) regulation of gut-derived lymphocytes; and (3) alteration of gut-associated metabolites. In addition, gut metabolites and bacteria from circulation may affect lymphocyte-mediated immune responses in the kidney. Therefore, they may also become therapeutic targets.

Gut microbiota depletion by oral antibiotics was proved to protect against IR-induced AKI in mice and the protective role was related to Th1 and Th17 decrease accompanied by Treg expansion in kidneys (Yang et al., 2020). The reduction of *Lactobacilli* was one of the hallmarks of IRI-induced gut microbial dysbiosis (Yang et al., 2020), and a similar decrease was discovered in CKD (Yang et al., 2019). Oral administration of *Lactobacilli* led to improvements in SCFAs (Vemuri et al., 2019). Therefore, supplementation of probiotics like *Lactobacilli* may be a promising strategy for AKI and CKD therapies. However, the following three questions remained to be solved: (1) Whether the reno-protective role of probiotics is achieved through the modulations of lymphocytes? (2) How do they act on lymphocytes? (3) What is the connection of lymphocyte alterations between the gut and kidneys? The potential therapeutic strategies of AKI and CKD based on the gut-kidney axis deserve further studies (shown in Figure 3).

## Conclusions

Lymphocytes mediate non-autoimmune AKI and subsequent CKD in a full-course manner. The diversity and plasticity of lymphocytes result in their multifunction for renal injury and fibrosis. Innate and adaptive lymphocytes cooperate in the process and present a phenomenon of chronological responses, which suggests the importance of the distinct “time windows” of therapies targeting different lymphocyte subsets. Interventions of lymphocytes *in vivo* and adaptive transfer of these cells have provided the prospect of lymphocyte-related therapeutic strategies. The modulations of gut microbiota and metabolites to regulate AKI- or CKD-associated lymphocyte immune responses show therapeutic potential for future drug development for kidney diseases. Despite many efforts made to explore the mechanisms of lymphocyte regulations to prevent AKI and subsequent renal fibrosis or promote renal repair, some problems remain to be addressed in the future: (1) What are the sources of the renal infiltrated lymphocytes, and what are the relationships between these lymphocytes and those of extrarenal organs? (2) How to achieve precise intervention on pathogenic lymphocytes without affecting systemic immunity? With the development of the technique of single-cell RNA sequencing, we are looking forward to a deeper understanding of lymphocyte subtypes and functions, which guide more precise and specific interventions to treat AKI and CKD.

## Author Contributions

CC: writing—original draft. YY: conceptualization and supervision. RZ: writing—reviewing and editing. All authors contributed to the article and approved the submitted version.

## Funding

This study was financially supported by the National Natural Science Foundation of China (Grant Nos: 81770681, 81974086, 81770684, and 81974087).

## Conflict of Interest

The authors declare that the research was conducted in the absence of any commercial or financial relationships that could be construed as a potential conflict of interest.

## Publisher's Note

All claims expressed in this article are solely those of the authors and do not necessarily represent those of their affiliated organizations, or those of the publisher, the editors and the reviewers. Any product that may be evaluated in this article, or claim that may be made by its manufacturer, is not guaranteed or endorsed by the publisher.
